# Ammonia gas sensors based on undoped and Ca-doped ZnO nanoparticles

**DOI:** 10.1039/d3ra08181h

**Published:** 2024-02-08

**Authors:** M. Hjiri, Saja Algessair, R. Dhahri, Hasan B. Albargi, N. Ben Mansour, A. A. Assadi, G. Neri

**Affiliations:** a Department of Physics, College of Sciences, Imam Mohammad Ibn Saud Islamic University (IMSIU) Riyadh 11623 Saudi Arabia mbhjiri@imamu.edu.sa m.hjiri@yahoo.fr +966-506163909; b Department of Physics, College of Sciences and Arts, Najran University P. O. Box 1988 Najran 11001 Saudi Arabia; c Laboratory of Physics of Materials and Nanomaterials Applied at Environment (LaPhyMNE), Faculty of Sciences in Gabes, Gabes University Gabes Tunisia; d College of Engineering, Imam Mohammad Ibn Saud Islamic University, IMSIU Riyadh 11432 Saudi Arabia; e Department of Engineering, University of Messina Messina 98166 Italy

## Abstract

Due to its large use in different industrial sectors, high toxicity, and corrosion, the demand for sensing techniques towards ammonia gas has become urgent. In this study we report on the sensing performances of a conductometric sensor for NH_3_ gas based on Ca-doped ZnO nanoparticles with different calcium concentrations (0, 1, and 3 at%) synthesized using the sol–gel process under supercritical dry conditions of ethanol. All samples were characterized using X-ray diffraction (XRD), scanning electron microscopy (SEM), transmission electron microscopy (TEM), and Fourier-transform infrared (FTIR) spectroscopy. Pure and Ca-doped ZnO are polycrystalline and well crystallized in the hexagonal wurtzite structure. TEM images revealed that pure ZnO is composed of spherical particles with dimensions in the nanometer range. Larger particles were observed after the incorporation of Ca ions. The average crystallite size, estimated by the Williamson–Hall method, was 43, 80, and 96 nm for pure, Ca-1 at% and Ca-3 at%, respectively. Furthermore, FTIR spectroscopy was used to prove the formation of ZnO and the incorporation of calcium ions in the Ca-doped ZnO samples. The gas sensing performances towards ammonia gas clearly ameliorated after the addition of Ca ions in the ZnO structure. The gas response to NH_3_, *R*_0_/*R*_g_, of the 1% Ca-doped ZnO sensor reached a value of 33 for 4000 ppm of ammonia at *T* = 300 °C with good selectivity compared to other gases such as CO, CO_2_, and NO_2_. The response and recovery times were 5 s and 221 s, respectively. The reported good sensing performances indicate the potential application of Ca-doped ZnO as a sensor material for ammonia detection.

## Abstract 2

Ca-ZnO gas sensor exibited a good response and high selectivity toward NH_3_ gas. Theses performances make the material an excellent candidate for monitoring ammonia gas at low concentrations.

## Graphical abstract

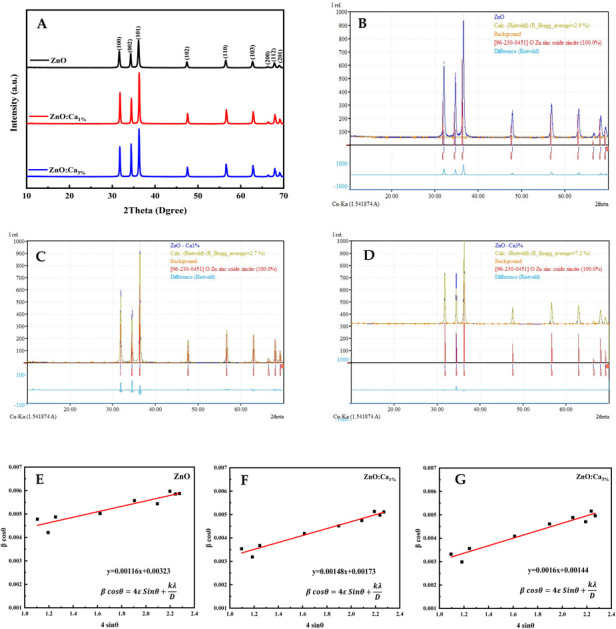

## 1. Introduction

Ammonia gas (NH_3_) is often used in the production of textiles, polymers, refrigerant systems, fertilizers, insecticides, explosives, and food-based products.^1–3^ It is a colorless poisonous gas with a strong odor that has a threshold limit of 25 ppm. The long-term inhalation of NH_3_ can be fatal^4,5^ since it can harm the eyes, skin, and the human respiratory system. In one instance, the American National Institute for Occupational Safety and Health specified that the immediately harmful to life or health concentration (IDLH) should be no more than 300 ppm. The Occupational Safety and Health Administration (OSHA) has established a threshold limit of 50 ppm for the workplace. So, to safeguard our clean environment and regularly monitor the leakage of this gas, it is crucial to create a gas sensor to detect ammonia gas.^6^

Since metal oxide materials offer great qualities, such as a high sensing response, long-term stability, and ease of synthesis, they may generally be employed for gas sensing detections.^7^ These types of materials adsorb oxygen molecules, which causes the oxygen to become ionized by abstracting an electron for the metal oxides conduction band, resulting in the production of a surface depletion layer, and consequently increasing resistance. SnO_2_, TiO_2_, ZnO,^8,9^ WO_3_, and Fe_2_O_3_ (ref. 10) are a few examples of several metal oxide-based gas sensors with diverse morphologies. ZnO nanostructures, which are among these metal oxides, have been widely employed as gas sensors because of their superior sensing response, strong selectivity, low cost, simple fabrication, outstanding thermal and chemical stability, and non-toxicity. In addition, ZnO material exhibited n-type conductivity having a resistivity in the range of 10^−4^ to 10^12^ Ω cm,^11^ broad bandgap (3.3 eV) at ambient temperature, high exciton binding energy (60 meV), strong electron mobility, great thermal stability, and non-toxicity.^12^

During the previous several decades, the number of industries using ammonia gas sensors has increased largely. In fact, there has been a significant increase in the interest in developing NH_3_ gas sensors, which should present key characteristics including good sensitivity, selectivity, stability, and reversibility. They also need to be safe, affordable, small, lightweight, and able to operate in a wide range of temperatures.^13,14^ For example, Kumar *et al.* synthesized MnO_2_ nanofibers and tested them toward 100 ppm of NH_3_ gas at ambient temperature. The fabricated sensors showed a response of 20% and response and recovery times of 200 and 100 s, respectively.^15^ Besides, the ammonia gas sensor based on SnO_2_ nanostructures fabricated by Beniwal and his team presented a response of 92% toward 500 ppm at ambient temperature and response/recovery times of 29/49 s.^16^ To develop ammonia gas sensor-based ZnO material with higher performances, numerous ways are utilized such as (i) applying an electrostatic field, (ii) using ultraviolet radiations in sensing operation, and (iii) doping materials using suitable metals.^17–23^

In this study, we selected calcium as a dopant of ZnO to improve the sensing properties of ammonia gas. Calcium is cheap and non-toxic to human health contrary to other elements. It is also the third most common metal in nature, after iron and aluminum. The incorporation of Ca^2+^ ions promotes the decrease of the near-band edge emission and the increase of oxygen vacancy defects, which could improve the sensing characteristics. A sol–gel method was used to fabricate ZnO and ZnO:Ca nanopowders. After performing the structural and morphological characterizations, the synthesized powders were tested for NH_3_ sensing. The sensing mechanism was probed and discussed. The results and information acquired allowed the development of a simple conductometric platform for ammonia gas detection with good sensitivity and selectivity.

## 2. Experiments section

### 2.1. Synthesis of ZnO:Ca nanopowders

A sol–gel method was used in the preparation of metal oxide nanomaterials because of the cheaper price, simple setup, and reduced energy use associated with this method. In this context, ZnO nanomaterials doped with Ca and with varying Ca contents (0, 1, and 3 at%) were synthesized supercritically under dry ethanol circumstances (critical temperature = 243 °C; critical pressure = 63.6 bars). 16 g of zinc salt [Zn(CH_3_COO)_2_·2H_2_O] and the calcium salt [CaCl_2_·6H_2_O] were dissolved in methanol solvent, as suggested by El Mir and his team.^24,25^ The drying process was performed in a stainless-steel autoclave. The obtained ZnO, ZnO:Ca_1%_ (C1ZO), and ZnO:Ca_3%_ (C3ZO) nanopowders were then heat-treated for two hours at 400 °C.

### 2.2. Samples characterization

#### XRD measurements

An X-ray diffractometer (AXS D8 Advance; BRUKER, Billerica, MA, USA), working at 40 kV voltage and 40 mA with copper Kα irradiation of wavelength of 0.154056 nm in the 2*θ* range from 20 to 80 2-theta degree, was used to study the nanopowder crystallographic structure. After being ground into a fine powder in a mortar, a small portion was placed in the sample holder to perform measurements.

#### Scanning electron microscopy (SEM)

The surface morphology of the samples was observed using a scanning electron microscope (JEOL 5600LV, Tokyo, Japan). Samples of nanoparticles were coated with 10 nm Pt (to stop the creation of electrostatic charges) and then attached to an SEM holder using carbon tape. SEM was powered by a 15 kV acceleration voltage.

#### Transmission electron microscopy (TEM)

Using a Tecnai G20-S-Twin transmission electron microscope (FEI, Eindhoven, The Netherlands) with a 200 kV accelerating voltage, the morphology of the nanopowders was investigated. A small quantity of nanopowdered samples was dissolved in ethanol using an ultrasonic bath (Branson CPX5800H-E, USA) for a few minutes. The resulting solution was then poured onto a Cu grid covered with carbon and allowed to dry at room temperature in a clean room.

#### Fourier transform infra-red (FTIR) spectroscopy

A PerkinElmer 100 Spectrum FTIR spectrophotometer was used to investigate the functional groups in the different samples. After cleaning up all apparatus with acetone to avoid any contamination, a small portion of the sample was placed in an agate mortar; then a KBr was added. The mixture was ground until a fine powder was obtained. Then, the sample was placed in the FTIR compartment for the measurement.

### 2.3. Sensing tests

Using alumina substrates (6 mm × 3 mm) and platinum interdigitated electrodes, thick films of ZnO and ZnO:Ca powders dispersed in water (1–10 m thick) were printed to fabricate the devices for the sensing tests. On the underside of the electrodes, there was a Pt heater. A synthetic dry air total stream of 100 sccm was used for the electrical experiments, which were conducted in the RT to 400 °C temperature range. The four-point method was used to obtain the resistance data. The gas sensing performances were evaluated using a flow-through method. A dual-channel power supplier instrument, Agilent E3632A, was utilized to bias the device's built-in heater while a multimeter data collecting unit, Agilent 34970A, was used to collect the data. In order to execute NH_3_ detection, pulses of this gas from certified bottles were injected into the 5 cc test chamber, at 100 sccm. Mass flow controllers and dry synthetic air (20% O_2_ in nitrogen) as the diluting carrier, were used to adjust the desired gas concentration from 0.025 to 0.4% (250 to 4000 ppm). The gas response, *S*, is given as *S* = [*R*_0_/*R*_g_], where *R*_0_ is the electrical resistance in synthetic air at the baseline and *R*_g_ is the electrical resistance of the sensor at various NH_3_ concentrations.

## 3. Results and discussions

### 3.1. Structural and morphological properties

**Fig. 1:**
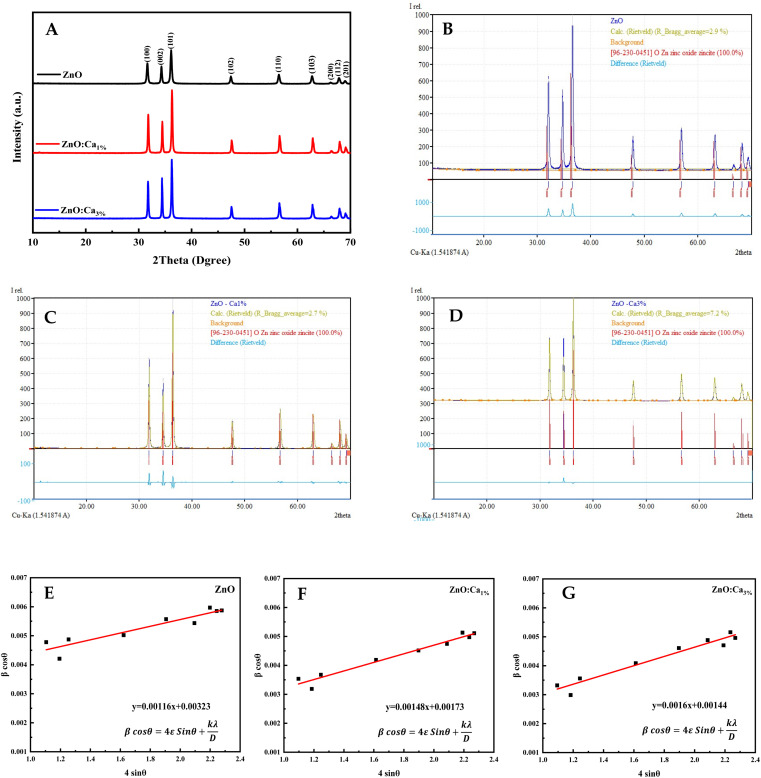
(A) X-ray diffraction of ZnO and ZnO:Ca NPs. (B–D) Rietveld refinement plots of ZnO, ZnO:Ca_1%_, and ZnO:Ca_3%_ (the blue curve represents the observed data, and the olive green color is the calculated pattern; the light blue curve shows the difference between the calculated and the observed intensities). (E–G) Williamson–Hall plots of ZnO, ZnO:Ca_1%_, and ZnO:Ca_3%_.

The XRD patterns of the ZnO, ZnO:Ca_1%_, and ZnO:Ca_3%_ samples are displayed in Fig. 1A. The observed diffraction peaks are indexed to the hexagonal wurtzite structure of ZnO (space group: *P*6_3_*mc* (186)) in agreement with (COD card no. 96-230-0451). The 2*θ* peaks appeared at 2*θ* ≈ 32.07°, 34.72°, 36.55°, 47.85°, 56.9°, 63.17°, 66.67°, 68.24°, and 69.4° corresponding to the crystal faces (100), (022), (101), (102), (110), (103), (200), (112) and (201), respectively.^26,27^ No extra peaks were observed due to the presence of any secondary phases.

The Rietveld refinement analysis shown in Fig. 1B and C was performed using the Match! software to obtain accurate structural parameters. The refined lattice parameters and other crystallographic data are summarized in Table 1. The lattice parameters “*a*” and “*c*” showed slight reductions upon calcium doping while the density increased, suggesting the successful incorporation of Ca into the ZnO lattice.

**Table 1:** The average crystallite sizes and parameters deduced from

Parameter		Samples		
		ZnO	ZnO:Ca_1%_	ZnO:Ca_3%_
*D*_s_ (nm)		26.55	32.84	33.78
*D*_WH_ (nm)		42.93	80.15	96.29
Microstrain *ε*		0.00116	0.00148	0.0016
Space group		*P*6_3_*mc* (186)	*P*6_3_*mc* (186)	*P*6_3_*mc* (186)
Crystal system		Hexagonal	Hexagonal	Hexagonal
Density (g cm^−3^)		5.671	5.678	5.679
Lattice parameters	*a* (Å)	3.2508	3.2495	3.2491
	*c* (Å)	5.2078	5.2058	5.2054

The crystallite sizes of all samples were determined using Scherrer's equation and Williamson–Hall's relation. Scherrer equation relates the crystallite size (*D*_s_) to the peak broadening (*β*), wavelength (*λ*), and Bragg angle (*θ*) of the X-ray radiation as follows:^28^
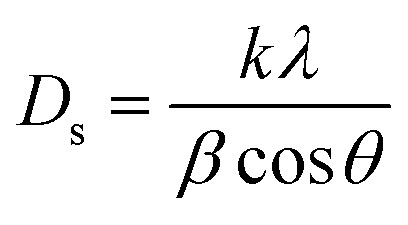
where *K* is the Scherrer constant (assumed as 0.9 for spherical particles).

Williamson–Hall's relation incorporates both the crystallite size (*D*_WH_) and microstrain (*ε*) effects on the peak broadening:^29^
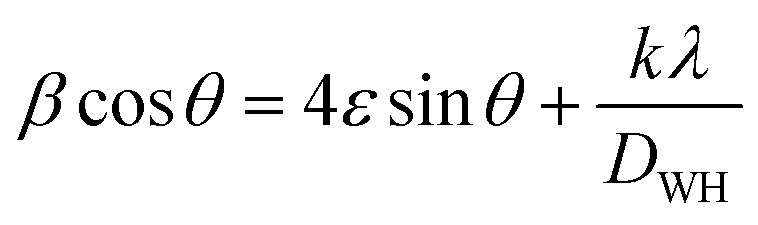


The calculated crystallite sizes using Scherrer's equation and Williamson–Hall's relation, along with the microstrain values, are also presented in Table 1. The comparison of the crystallite sizes obtained from Scherrer's equation and Williamson–Hall's relation revealed interesting trends. For all the samples, both methods indicate an increase in the crystallite size with calcium doping. The ZnO:Ca_3%_ sample exhibited the largest crystallite size, followed by ZnO:Ca_1%_ and ZnO. Comparing the two methods, the Scherrer equation generally yields smaller crystallite sizes than the Williamson–Hall's relation. This discrepancy can be attributed to the inclusion of the microstrain effects in the latter. The presence of microstrain can arise from lattice defects, dislocations, or strain induced by the doping process.^29^ The larger crystallite sizes obtained using Williamson–Hall's plot (Fig. 1E–G) indicate the presence of microstrain in the samples. Moreover, the microstrain also increased with an increase in calcium content, indicating the generation of more defects due to doping.

**Fig. 2:**
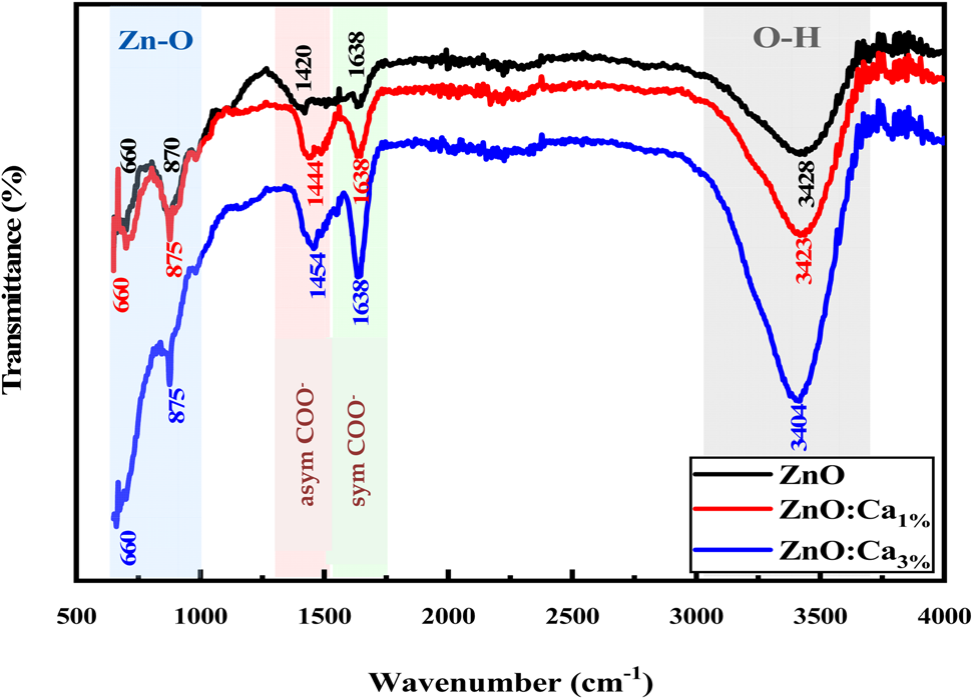
FTIR spectra of ZnO and ZnO:Ca NPs.

From FTIR, we investigated the functional groups in the undoped and Ca-doped samples. The spectra were recorded in the range of 500–4000 cm^−1^. FTIR spectra of all samples, shown in Fig. 2 exhibit several characteristic absorption bands.

For undoped ZnO, the broad peak around 3100–3700 cm^−1^ corresponds to the stretching vibration of O–H bonds in the hydroxyl groups (–OH) present on the surface of ZnO nanoparticles or adsorbed moisture.^30^ The peaks around 1420 and 1638 cm^−1^ are due to the production of carbonate species^31,32^ and the specifics related to asymmetric and symmetric COO^−^ vibrations. In addition, the sharp peaks at 650 cm^−1^ and 870 cm^−1^ correspond to Zn–O stretching vibrations, which is a characteristic feature of ZnO.^33^ When calcium is doped into the ZnO lattice, changes in the intensity of the O–H stretching peak around 3200–3700 cm^−1^ were noted. The increase in the intensity suggests an increase in the concentration of O–H groups, likely due to the dopant calcium ions, which promote the adsorption of water. Further, whereas no intensity changes of the peaks in the fingerprint region (500–1000 cm^−1^) for ZnO and ZnO:Ca_1%_ was observed; with ZnO:Ca_3%_, there is an increase of the peak intensity in this region, due to the higher amount of calcium in the ZnO:Ca_3%_ sample. In any case, the presence of this impurity was not detectable by FTIR because the Ca–O bond overlaps with the Zn–O spectral bands in that region.^18^

**Fig. 3:**
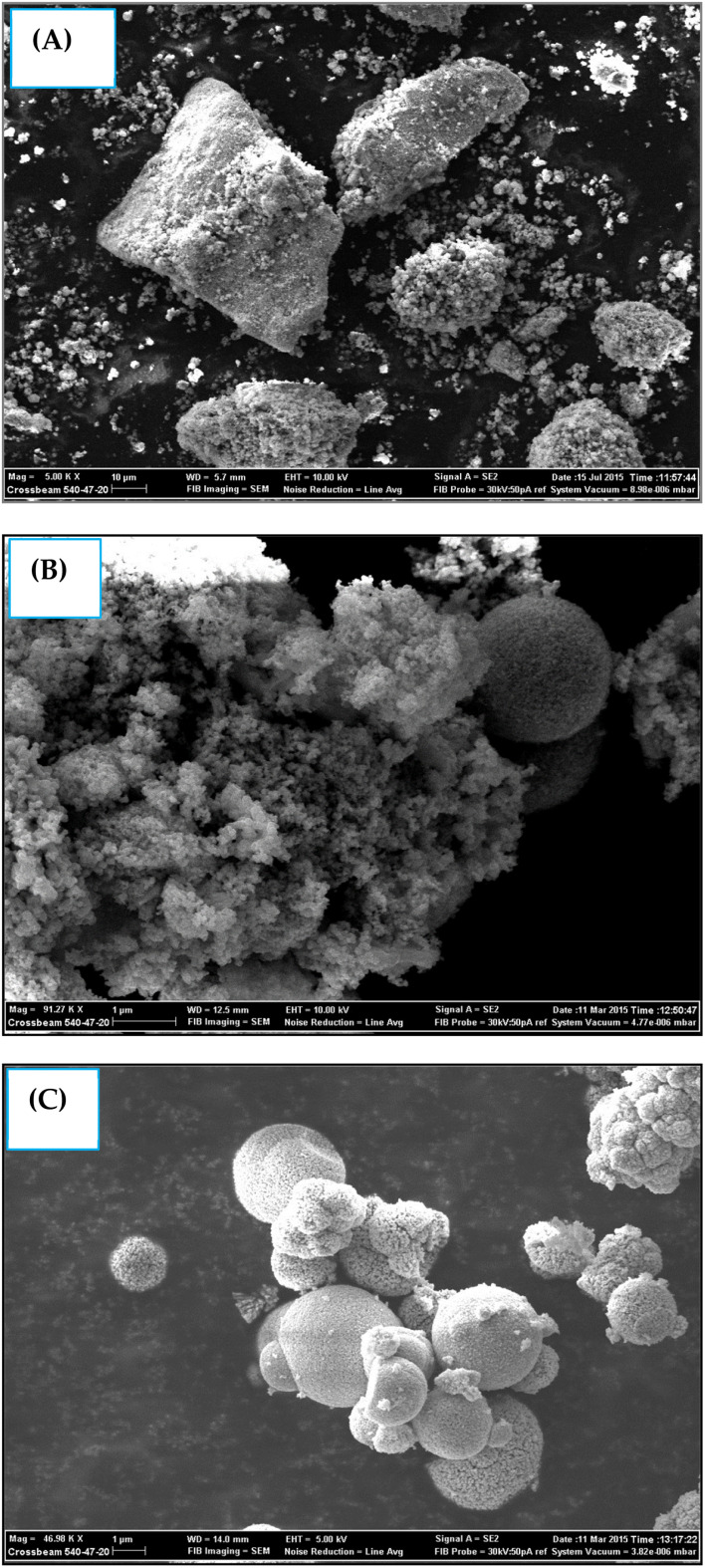
SEM photographs of (A) pure ZnO, (B) ZnO:Ca_1%_, and (C) ZnO:Ca_3%_ nanoparticles.

Fig. 3A–C shows the surface morphology images of the samples recorded through SEM analysis. All the synthesized samples appeared to be composed of small nanoparticles that were highly agglomerated.

**Fig. 4:**
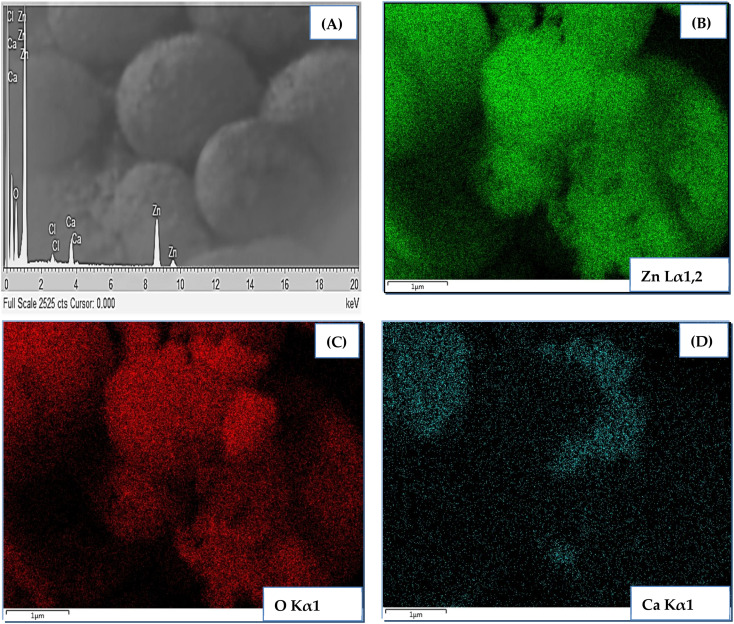
EDS spectrum and elemental color mapping of 1 at% CZO.

Elemental analysis of CZO (1 at%) was carried out using color mapping analysis and EDX. The outcomes shown in Fig. 4 confirmed the presence of main elements, *i.e.*, Zn, O, and Ca in the sample with good uniform distribution. No meaningful presence of any other contaminating species was recorded.

**Fig. 5:**
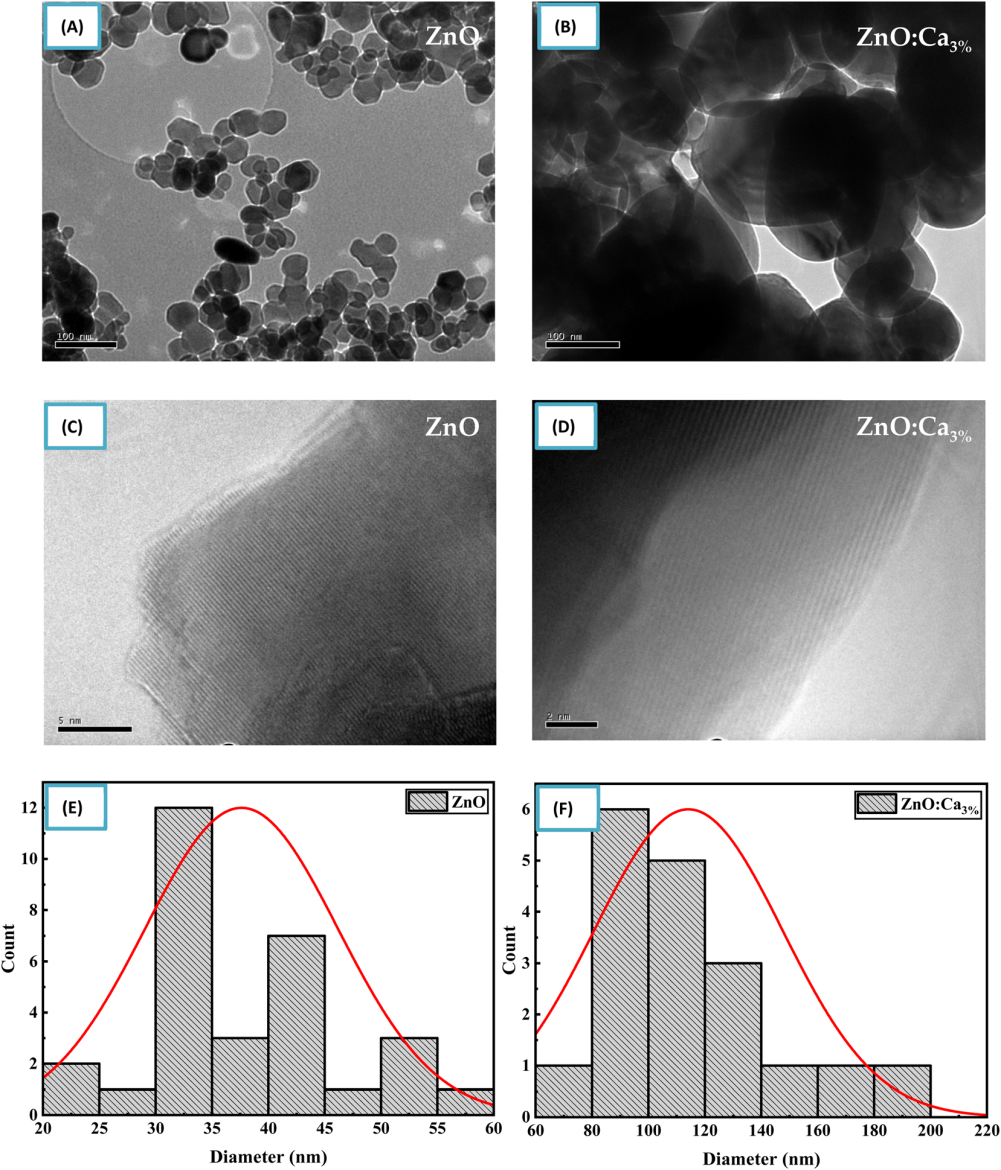
(A–D) TEM and HRTEM images of ZnO and ZnO:Ca nanoparticles. (E and F) Particle size distribution of ZnO and ZnO:Ca nanoparticles.

Fig. 5(A) and (B) shows TEM and (C) and (D) HRTEM images of ZnO and ZnO:Ca_3%_ samples. The images clearly show that the doped and undoped NPs have asymmetric spherical-like shapes and clearly visible lattice fingers.

The particle size distribution was determined from the TEM image using an image analysis software by measuring the diameter of individual nanoparticles and creating a histogram showing the frequency of nanoparticles at each diameter size as shown in Fig. 5(E) and (F). The size distribution illustrated that the mean particle sizes of ZnO and ZnO:Ca_3%_ are 37.62 nm, and 114.15 nm respectively. This confirmed the increase in particle size with calcium doping, supporting the XRD findings.

### 3.2. Sensing performances

**Fig. 6:**
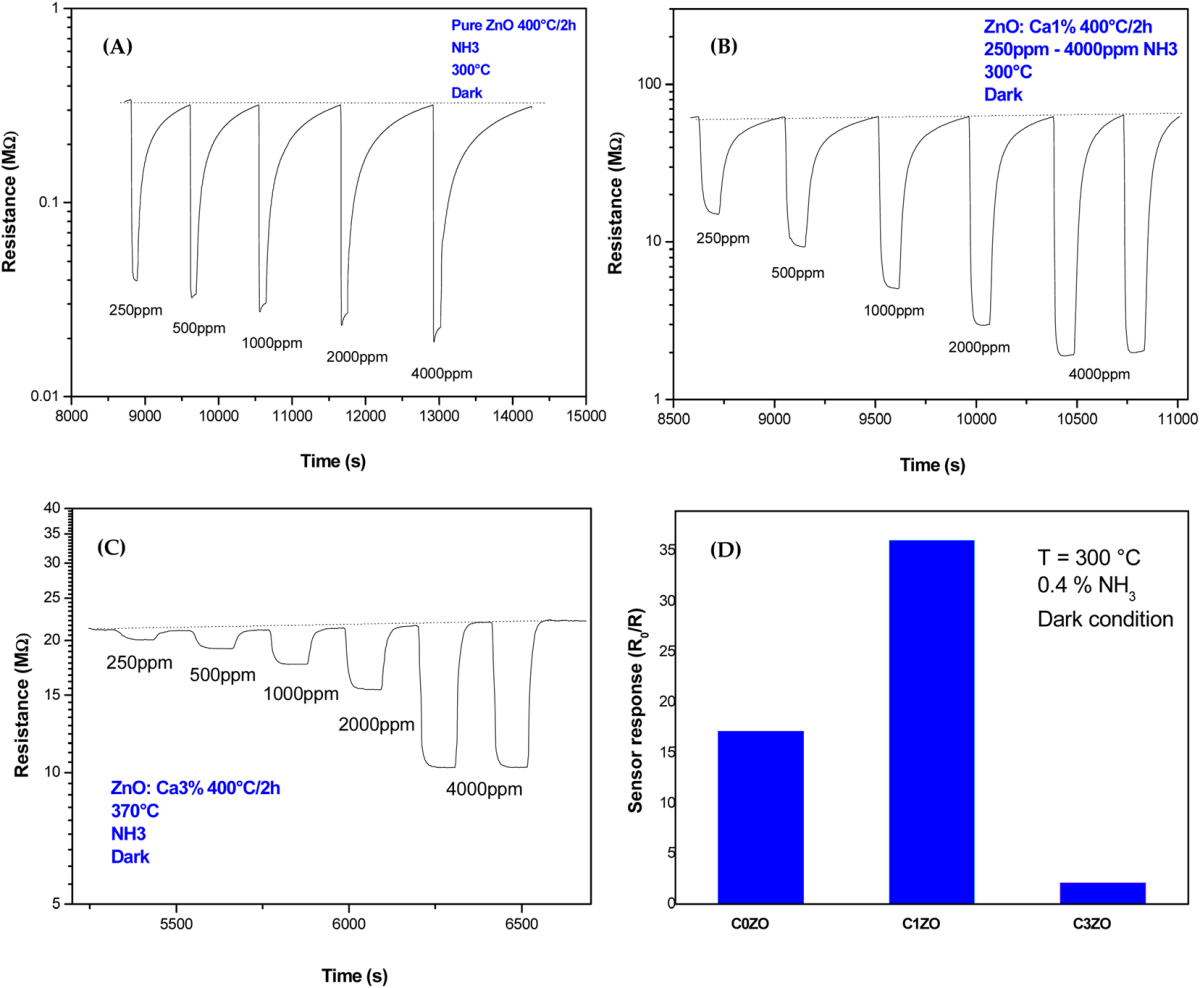
Dynamic response of (A) pure ZnO, (B) ZnO:Ca_1%_, and (C) ZnO:Ca_3%_ nanoparticles toward various concentrations of NH_3_ gas. (D) Response of different samples toward 0.4% of NH_3_ at *T* = 300 °C.

Dynamic responses of different samples are reported in Fig. 6. The operating temperature was 300 °C and the samples were tested for various concentrations of NH_3_ gas. In all the samples, the resistance was decreased after the exposure of the gas, and then it returned to the starting value indicating that ZnO material kept its n-type nature with NH_3_ injection. This was normal behavior because ammonia is a reducing gas and Ca dopant did not alter the n-type nature of ZnO. The same results were found by Radhi Devi and the team when the strontium-doped ZnO sensor was tested toward various concentrations of NH_3_ gas.^34^ Kathwate *et al.* also noticed that the resistance of the Al-doped ZnO films decreased after ammonia injection.^35^ In contrast, Nakarungsee's team noticed that the resistance increased when Cr-doped ZnO sensors were tested toward NH_3_ gas.^36^ The decrease of material resistance could be explained using the traditional theory of resistance change^37^ following the contact with a reducing molecule on the surface of the n-type semiconductors, which may be used to understand the observed drop in resistance. The resistance decrease was a result of gas molecules and adsorbed oxygen species interactions. The adsorbed oxygen species and reducing gas molecules will react on the surface of the material as described by the reaction below:2NH_3_ + 10O^−^ → 2NO + 3H_2_O + 10e^−^

The resistance drop phenomenon after the NH_3_ (reducing gas) injection was observed in ZnO and Ca-doped sensors suggesting that the incorporation of calcium ions did not alert the n-type nature of the ZnO material.

For pure ZnO sensors, the baseline resistance takes a value of 0.32 MΩ. A small change in resistance is noticed with NH_3_ concentrations increase, as shown in Fig. 6(A). In Fig. 5(C) and 6(B), Ca-doped sensors exhibited a bigger baseline resistance (60 MΩ for C1ZO and 22 MΩ for C3ZO) in addition to a remarkable change in the resistance after the gas injection for both doped samples. The baseline resistance of doped samples was higher than that of the undoped ones. The same behavior was shown by Dhahri *et al.* in their research;^38^ Misra's team^39^ has also observed an increase in the baseline resistance in Ca-doped material. This is probably due to a drop in the concentration of carriers and/or due to the creation of a carbonate layer after the calcium incorporation in the ZnO network, which changes the semiconductor surface.^40^

As displayed in Fig. 6(D) the response, calculated as, 
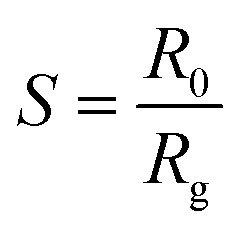
^41^ reached a maximum value of 33 for the C1ZO sensor toward 0.4% NH_3_ concentration at *T* = 300 °C, and it was reduced 10 times for the C3ZO sample. This response reduction for the C3ZO sensor can be explained as follows: at higher doping levels, calcium ions may not substitute zinc ions but may be going into the interstitial sites inside the ZnO or on the surface. In addition, as mentioned above ZnO:Ca_1%_ exhibited a particle size smaller than that of ZnO:Ca_3%_. Smaller crystallite size means higher surface area and more sensitive sites. In addition, on the ZnO:Ca_3%_ sample, there is an excess of Ca that covers the zinc oxide surface and decreases the interaction sites, consequently leading to a reduction of gas response.

**Fig. 7:**
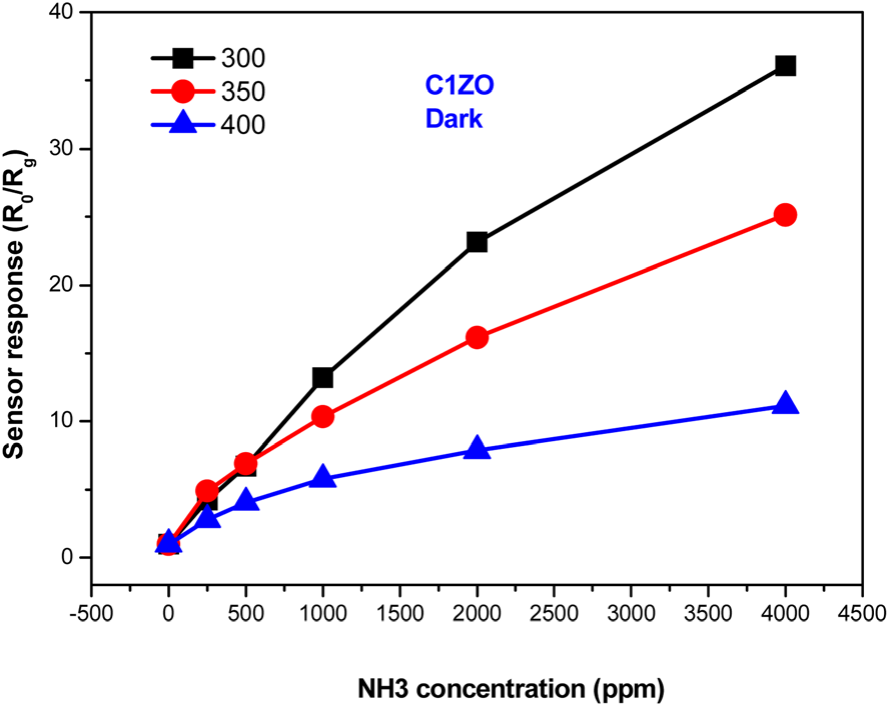
Variation of the ZnO:Ca_1%_ gas response towards different ammonia concentrations at different temperatures.

Fig. 7 shows the response of the ZnO:Ca_1%_ material toward different ammonia concentrations at various temperatures. It is clearly seen that at low NH_3_ concentrations, the gas response increases slowly upon reducing the temperature. After increasing gas concentrations, the response enhances largely and reaches higher values at *T* = 300 °C. We can say that the greatest response toward ammonia was obtained at lower temperatures (*T* = 300 °C).

**Fig. 8:**
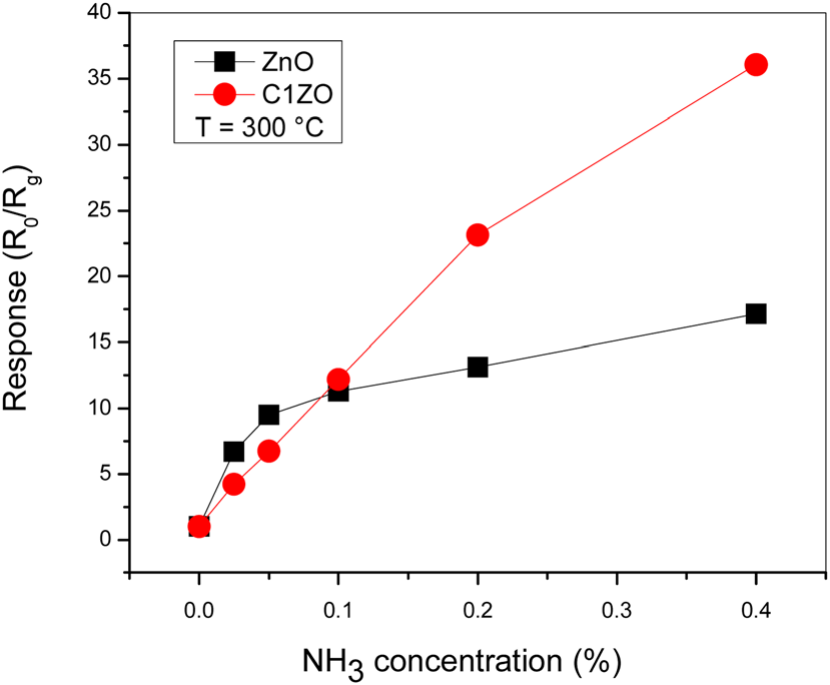
Comparison between pure ZnO and ZnO:Ca_1%_ sensors response toward different NH_3_ concentrations at *T* = 300 °C.

The gas response of the undoped and ZnO:Ca_1%_ sensors toward different ammonia concentrations at *T* = 300 °C is presented in Fig. 8. At low gas concentrations, the undoped sensor exhibits a higher response compared to the doped one. Increasing the gas concentrations, the C1ZO sensor response enhances and becomes better than that of the pure sensor until reaches a maximum value of *S* = 33. This behavior can be explained in terms of electronic defects such as oxygen vacancies (V_O_) and zinc interstitials (Zn_i_), which are created after the addition of Ca ions in the ZnO network, as cited by Saeedi and his colleagues.^42^ Zn_i_ and V_O_ acted as donor defects, leading to an increase in the concentration of free electrons.^43^ These free electrons take part in the interaction between the sensing layer and ammonia molecules by reducing C1ZO sensing layer resistance and then the response increases. Besides, ammonia adsorption is favored on zinc active centers. However, increasing the concentration of ammonia, leads to the saturation of these active centers, while the presence of Ca ions can maintain the surface cleaner, favoring the desorption of ammonia from the Ca–ZnO active sites. This was confirmed by the very large difference in the recovery time, which is very long for the pure ZnO and becomes faster increasing the Ca loading.

**Fig. 9:**
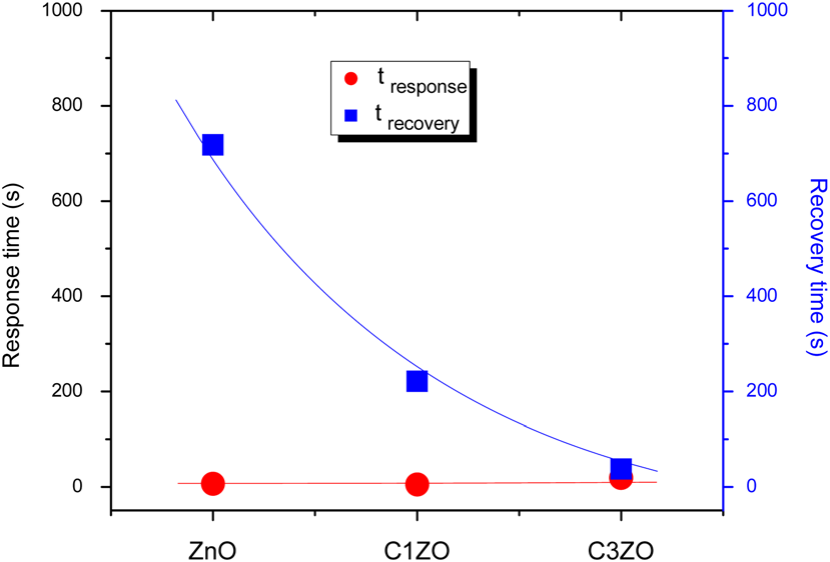
Response/recovery times of various sensors.

The response/recovery times of different sensors are displayed in Fig. 9. The response/recovery times are (6 s, 718 s), (5 s, 221 s), and (18 s, 37 s) for pure ZnO, C1ZO, and C3ZO samples, respectively. All the samples exhibited quick response times, but slow recovery times, especially ZnO and C1ZO. This outcome showed that there was a considerable quantity of ammonia adsorbing on the sensing layer's surface, leading to the hypothesis that the rate at which ammonia or its intermediary species desorb from the surface is slower than the rate at which ammonia is adsorbed.^44^ The recovery time of the C3ZO sensor seems to be faster.

**Fig. 10:**
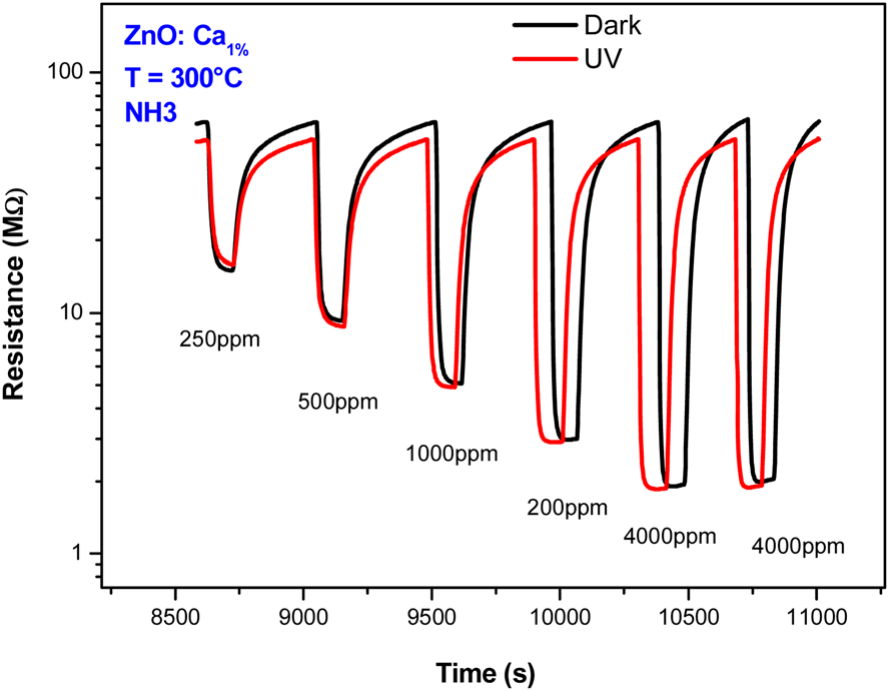
Dynamic response behaviors of ZnO:Ca_1%_ sensor toward NH_3_ concentrations with and without UV light illumination.

Also, the effect of UV light was investigated. Fig. 10 presents the dynamic response of the C1ZO sensor toward different concentrations of ammonia at *T* = 300 °C under dark conditions and UV radiation. Under UV light illumination, the baseline resistance of the sensing layer decreased a bit. The drop in the resistance of metal oxides by UV light is due to photoelectron activation. In fact, a decrease in the indium oxide resistance model suggested by Wagner's team highlighted the diffusion of oxygen species in and out of the In_2_O_3_ lattice after radiation with 350 nm.^45^ The sensor response is also little influenced by the presence of the ultra-violet light. This behavior can be explained by considering that operating with UV at high temperatures generally does not lead to an improvement of the sensing properties. The positive influence of ultra-violet illumination on electrical and gas sensing properties is instead well recognized at low operating temperatures.

**Fig. 11:**
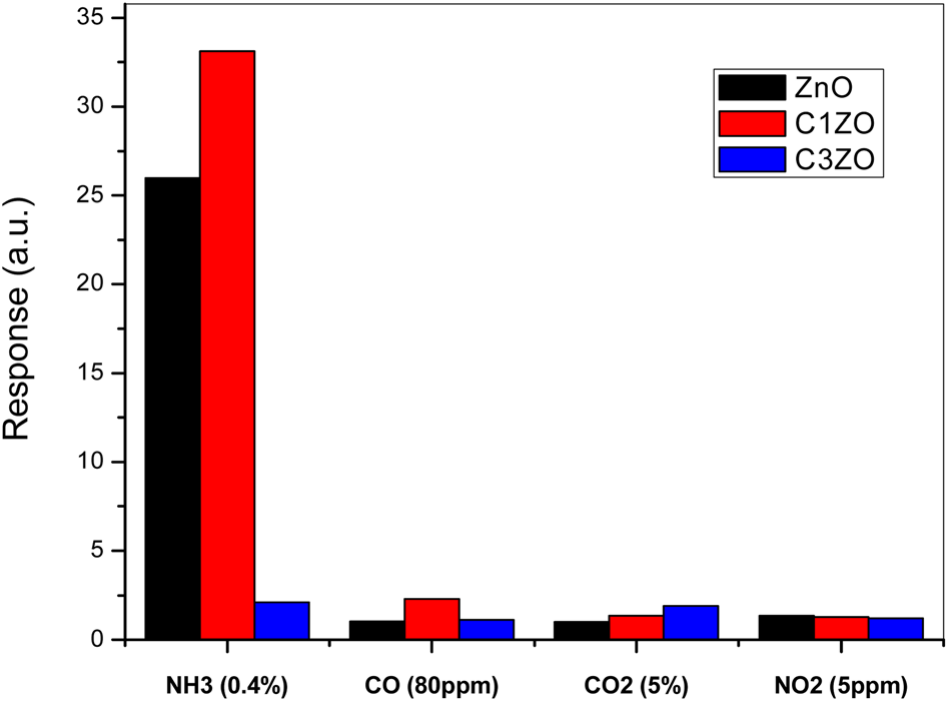
Selectivity of the studied sensors when tested toward a mixture of gases (NH_3_, CO, CO_2_, and NO_2_).

To show the high selectivity of the suggested sensors, especially C1ZO one, toward ammonia gas, all sensors were tested toward several oxidizing and reducing gases such as CO, CO_2_, and NO_2_, as shown in Fig. 11. In the beginning, the addition of 1 at% of calcium well improved the sensor response toward NH_3_. More calcium concentration (3 at%) in the ZnO network leads to poorer response compared to undoped and C1ZO sensors. The particle shape was excluded from the reasons for the gas sensor's response amelioration because all the samples maintained the same shape even after the incorporation of Ca^2+^ ions.

Testing sensors toward other gases (CO, CO_2_, and NO_2_) had a negligible effect on the gas response in comparison with ammonia indicating the high selectivity of the fabricated sensors for ammonia. Chaudhary and his team attributed this high selectivity to the smaller kinetic diameter (0.26 nm) and low ionization energy (10.18 eV) of ammonia.^46^ With its small kinetic diameter, NH_3_ can easily enter pores of the sensing layer and this leads to an enhancement of the gas response toward NH_3_.^46^

### 3.3. NH_3_ sensing mechanism

**Fig. 12:**
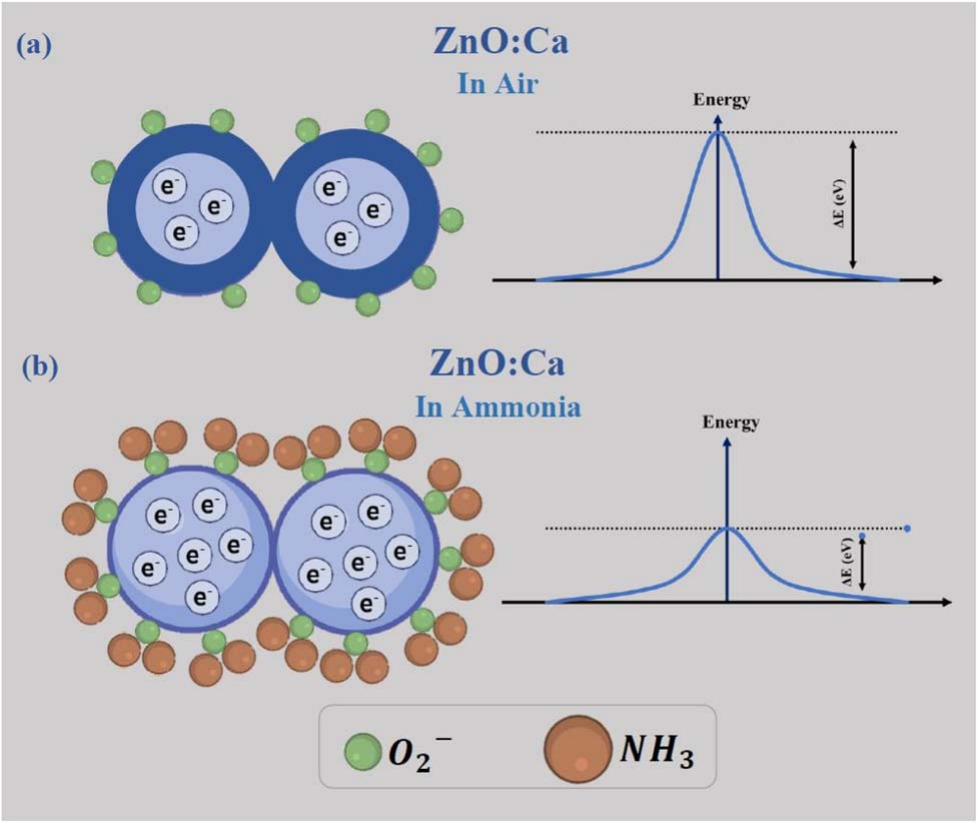
The sensing mechanism of ZnO:Ca_1%_ nanopowders (a) in air and (b) in ammonia atmosphere.

The chemical processes that occur between the sensing material and the target gas are at the basis of the ammonia gas sensing mechanism on metal oxide-based conductometric sensors.^47^ As observed in Fig. 12, two main steps describe these chemical processes: the first step is the exposure of the ZnO:Ca layer to air (baseline condition) when the adsorption of oxygen molecules occurs on the sensing surface. Adsorbed oxygen interacts with free electrons from the conduction band of the Ca-doped ZnO sensing material, leading to the creation of ionized oxygen species (O^2−^, O^−^, O_2_^−^), and the creation of a depletion layer on the surface and a potential barrier at the grain boundaries, establishing the baseline resistance (Fig. 12a).

In the second step, when NH_3_ is injected, the gas molecules are adsorbed on the surface and react with the adsorbed oxygen species, liberating free electrons. This process involves a decrease of the barrier potential (Fig. 12b), so the free electrons can flow easily, and consequently, a noticeable drop in the resistance was obtained in response to NH_3_ exposure, according to our experimental observations.

## 4. Conclusions

ZnO and ZnO:Ca thick films were screen printed on Pt-interdigitated alumina substrates and tested toward various concentrations of ammonia gas from 0.025–0.4%. The synthesized nanopowders were characterized by XRD, FTIR, TEM, and SEM. The samples were crystalline and showed the hexagonal wurtzite structure. The sizes of the typical crystallite, as determined by the Williamson–Hall method, increased with the addition of Ca. The gas sensing performances were clearly improved with the ZnO sensor doped with 1% of Ca. The developed sensor also displayed high NH_3_ selectivity with relation to other gases such as CO, CO_2_, and NO_2_, and fast response, thus demonstrating the possible use of ZnO:Ca for ammonia gas detection.

## Data availability

The data used to support the findings of this study are available from the corresponding author upon request.

## Author contributions

Mokhtar Hjiri: writing and editing; investigation; Saja Algessair: conceptualization; R. Dhahri: formal analysis; Hassan B Albargi: formal analysis; Nabil Ben Mansour: data curation; Amine Aymen Assadi: conceptualization; Giovanni Neri: supervision.

## Conflicts of interest

The authors declare no conflict of interest.

## Supplementary Material

## References

[cit1] Mani G. K., Rayappan J. B. B. (2015). A highly selective and wide range ammonia sensor—nanostructured ZnO: Co thin film. Mater. Sci. Eng., B.

[cit2] Poloju M., Jayababu N., Reddy M. R. (2018). Improved gas sensing performance of Al doped ZnO/CuO nanocomposite based ammonia gas sensor. Mater. Sci. Eng., B.

[cit3] Kwak D., Lei Y., Maric R. (2019). Ammonia gas sensors: a comprehensive review. Talanta.

[cit4] Shao F., Hernandez-Ramirez F., Prades J. D., Morante J. R., Lopez N. (2012). Assessment and modeling of NH3-SnO2 interactions using individual nanowires. Procedia Eng..

[cit5] Shahabuddin M., Sharma A., Kumar J., Tomar M., Umar A., Gupta V. (2014). Metal clusters activated SnO2 thin film for low level detection of NH3 gas. Sens. Actuators, B.

[cit6] Liu I.-P., Chang C.-H., Chou T. C., Lin K.-W. (2019). Ammonia sensing performance of a platinum nanoparticle-decorated tungsten trioxide gas sensor. Sens. Actuators, B.

[cit7] Bai S., Li D., Han D., Luo R., Chen A., Chung C. L. (2010). Preparation, characterization of WO3–SnO2 nanocomposites and their sensing properties for NO2. Sens. Actuators, B.

[cit8] Zhang Q., Xie G., Xu M., Su Y., Tai H., Du H., Jiang Y. (2018). Visible light-assisted room temperature gas sensing with ZnO-Ag heterostructure nanoparticles. Sens. Actuators, B.

[cit9] Wang S., Tai H., Liu B., Duan Z., Yuan Z., Pan H., Su Y., Xie G., Du X., Jiang Y. (2019). A facile respiration-driven triboelectric nanogenerator for multifunctional respiratory monitoring. Nano Energy.

[cit10] Sun P., Wang W., Liu Y., Sun Y., Ma J., Lu G. (2012). Hydrothermal synthesis of 3D urchin-like α-Fe2O3 nanostructure for gas sensor. Sens. Actuators, B.

[cit11] Mirzaei A., Janghorban K., Hashemi B., Bonyani M., Leonardi S. G., Neri G. (2016). A novel gas sensor based on Ag/Fe2O3 core-shell nanocomposites. Ceram. Int..

[cit12] Van De Pol F. C. (1990). Thin film ZnO-properties and applications. Am. Ceram. Soc. Bull..

[cit13] Timmer B., Olthuis W., Van Den Berg A. (2005). Sens. Actuators, B.

[cit14] Giddey S., Badwal S. P. S., Kulkarni A. (2013). Int. J. Hydrogen Energy.

[cit15] Kumar R., Kumar R., Kushwaha N., Mittal J. (2016). Ammonia gas sensing using thin film of MnO_2_ nanofibers. IEEE Sens. J..

[cit16] Beniwal A., Srivastava V. (2019). Sunny, Sol-gel assisted nano-structured SnO_2_ sensor for low concentration ammonia detection at room temperature. Mater. Res. Express.

[cit17] Zhang Y., Kolmakov A., Lilach Y., Moskovits M. (2005). J. Phys. Chem. B.

[cit18] Kolmakov A., Chen X. H., Moskovits M. (2008). J. Nanosci. Nanotechnol..

[cit19] Ramesh A., Gavaskar D. S., Nagaraju P., Duvvuri S., Vanjari S. R. K., Subrahmanyam C. (2022). Appl. Surf. Sci. Adv..

[cit20] Abdulsattar M. A., Jabbar R. H., Abed H. H., Abduljalil H. M. (2021). Optik.

[cit21] Brahma S., Huang P. C., Mwakikunga B. W., Saasa V., Akande A. A., Huang J. L. (2023). Mater. Chem. Phys..

[cit22] Song H., Ma L., Pei S., Dong C., Zhu E., Zhang B. (2021). Sens. Actuators, A.

[cit23] Ganesh R. S., Durgadevi E., Navaneethan M., Patil V. L., Ponnusamy S., Muthamizhchelvan C., Kawasaki S., Patil P. S., Hayakawa Y. (2018). Sens. Actuators, A.

[cit24] El Mir L., El Ghoul J., Alaya S., Salem M. B., Barthou C., Von Bardeleben H. (2008). Synthesis and luminescence properties of vanadium-doped nanosized zinc oxide aerogel. Phys. B.

[cit25] El Mir L. (2017). Luminescence properties of calcium doped zinc oxide nanoparticles. J. Lumin..

[cit26] Mahalakshmi S., Hema N., Vijaya P. P. (2020). In vitro biocompatibility and antimicrobial activities of zinc oxide nanoparticles (ZnO NPs) prepared by chemical and green synthetic route—a comparative study. Bionanoscience.

[cit27] Istrate A.-I., Nastase F., Mihalache I., Comanescu F., Gavrila R., Tutunaru O., Romanitan C., Tucureanu V., Nedelcu M., Müller R. (2019). Synthesis and characterization of Ca doped ZnO thin films by sol–gel method. J. Sol-Gel Sci. Technol..

[cit28] Hjiri M., Alonizan N. H., Althubayti M., Alshammari S., Besbes H., Aida M. S. (2019). Preparation and photoluminescence of NiFe_2_O_4_ nanoparticles. J. Mater. Sci.: Mater. Electron..

[cit29] Lemine O. M., Faqih A., Algessair S., Madkhali N., Hjiri M., Abu Alrub S., Alanazi A. Z., Alromaeh A., Mir L. (2023). Effect of Magnesium Ion Substitution on Physical Properties and Magnetic Induction Heating of Maghemite (γ-Fe2O3) Nanoparticles. J. Supercond. Novel Magn..

[cit30] Nipane D., Thakare S., Khati N. (2013). Synthesis of novel ZnO having cauliflower morphology for photocatalytic degradation study. J. Catal..

[cit31] Gu W., Bousfield D. W., Tripp C. P. (2006). Formation of calcium carbonate particles by direct contact of Ca(OH)_2_ powders with supercritical CO_2_. J. Mater. Chem..

[cit32] Prim A., Pellicer E., Rossinyol E., Peiró F., Cornet A., Morante J. R. (2007). Adv. Funct. Mater..

[cit33] Crispi S., Neri G. (2022). Development of a Conductometric Sensor Based on Al, Ca-Doped ZnO for the Detection of Formaldehyde. Sensors.

[cit34] Devi K. R., Selvan G., Karunakaran M., Raj I. L. P., Ganesh V., AlFaify S. (2020). Enhanced room temperature ammonia gas sensing properties of strontium doped ZnO thin films by cost-effective SILAR method. Mater. Sci. Semicond. Process..

[cit35] Kathwate L., Umadevi G., Kulal P., Nagaraju P., Dubal D., Nanjundan A., Mote V. (2020). Ammonia gas sensing properties of Al doped ZnO thin films. Sens. Actuators, A.

[cit36] Nakarungsee P., Srirattanapibul S., Issro C., Tang I.-M., Thongmee S. (2020). High performance Cr doped ZnO by UV for NH3 gas sensor. Sens. Actuators, A.

[cit37] Santangelo S., Fazio E., Neri F., Faggio G., Messina G., Neri G. (2013). Microstructure of anatase-based hybrid nanocomposites. J. Phys. D: Appl. Phys..

[cit38] Dhahri R., Hjiri M., El Mir L., Fazio E., Neri F., Barreca F., Donato N., Bonavita A., Leonardi S. G., Neri G. (2015). ZnO: Ca nanopowders with enhanced CO2 sensing properties. J. Phys. D: Appl. Phys..

[cit39] Misra K. P. , Dubey K., Shukla R. and Srivastava A., Reduction in carrier concentration by calcium doping in ZnO thin films, in 2009 International Conference on Emerging Trends in Electronic and Photonic Devices & Systems, IEEE, 2009, pp. 495–496

[cit40] Hong H. S., Dai Lam T., Trung T., Van Hieu N. (2012). Selective detection of carbon dioxide using LaOCl-functionalized SnO2 nanowires for air-quality monitoring. Talanta.

[cit41] Sivalingam D., Gopalakrishnan J. B., Rayappan J. B. B. (2012). Structural, morphological, electrical and vapour sensing properties of Mn doped nanostructured ZnO thin films. Sens. Actuators, B.

[cit42] Saeedi A. M., Alonizan N. H., Alsaigh A. A., Alaya L., El Mir L., El-Readi M. Z., Hjiri M. (2023). Phys. Status Solidi A.

[cit43] Janotti A., Van de Walle C. G. (2009). Rep. Prog. Phys..

[cit44] Fazio E., Hjiri M., Dhahri R., El Mir L., Sabatino G., Barreca F., Neri F., Leonardi S. G., Pistone A., Neri G. (2015). Ammonia sensing properties of V-doped ZnO: Ca nanopowders prepared by sol–gel synthesis. J. Solid State Chem..

[cit45] Wagner T., Kohl C. D., Morandi S., Malagù C., Donato N., Latino M., Neri G., Tiemann M. (2012). Chem.–Eur. J..

[cit46] Chaudhary D. K., Maharjan Y. S., Shrestha S., Maharjan S., Shrestha S. P., Joshi L. P. (2022). J. Phys. Sci..

[cit47] Neri G. (2015). First fifty years of chemoresistive gas sensors. Chemosensors.

